# Supplementary material for: Molecular Identification and Engineering a Salt-Tolerant GH11 Xylanase for Efficient Xylooligosaccharides Production
Source: Biomolecules. 2024 Sep 20;14(9):1188. doi: 10.3390/biom14091188 (PMC11430584; doi:10.3390/biom14091188)
Supplement: Supplementary file 1 [file biomolecules-14-01188-s001.zip › biomolecules-3182095-supplementary.pdf]

Supplementary Information

Molecular identification and engineering a novel salt-tolerant GH11 xylanase for efficient xylooligosaccharides production

Jiao Ma[1, 2], Zhongke Sun[1, 2, *], Zifu Ni[1], Yanli Qi[1], Qianhui Sun[1], Yuansen Hu[1, 2], Chengwei Li[1, 3, *]

1. School of Biological Engineering, Henan University of Technology, Zhengzhou, 450001, China
2. Food Laboratory of Zhongyuan, Luohe, 462333, China
3. College of Life Science, Henan Agriculture University, Zhengzhou, 450046, China

*, corresponding authors

Prof. Zhongke Sun, Email: sunzh@daad-alumni.de;

ORCID: https://orcid.org/0000-0002-9784-9769;

Prof. Chengwei Li, Email: lcw@haut.edu.cn; Tel: 86-0371-67756898

Post address: No.100, Lianhua Road, Gaoxin District, Zhengzhou City, Henan Province, 450001, China

Table S1 PCR primers used for amplification of the target genes

Primers	Sequence (5'-3')	Target/mutation site
P-XR	cgagctccgtcgacaagctt TTACCACACTGTTACGTTAG	Xynst
P-XF	tggtggtggtggtgctcgag ATGTTTAAGTTTACAAAG AAATTC	Xynst
Y5-F	GCTAACACAGACNNKTGGCAAAATTGGACTGATG	Tyr 5
Y5-R	CCATCAGTCCAATTTTGCCAMNNGTCTGTGTTAG	Tyr 5
W6-F	GCTAACACAGACTACNNKCAAAATTGGACTGATGG	Trp 6
W6-R	CATCAGTCCAATTTTGMNNGTAGTCTGTGTTAG	Trp 6
Q7-F	AACACAGACTACTGGDBBAATTGGACTGATGG	Gln7
Q7-R	CCATCAGTCCAATTVVHCCAGTAGTCTGTGTT	Gln7
W9-F	CAGACTACTGGCAAAATNNKACTGATGGGGGCGGAAC	Trp 9
W9-R	TTCCGCCCCCATCAGTMNNATTTTGCCAGTAGTCTGTG	Trp 9
R49-F	ACAGGTTCGCCATTTNNKACGATAAACTATAATGC	Arg 49
R49-R	CATTATAGTTTATCGMNNTAAATGGCGAACCTGTAG	Arg 49
Y65 F	CCGAATGGCAATGCANNKTTGACTTTATATGGTTGG	Tyr 65
Y65 R	AACCATATAAAGTCAAMNNTGCATTGCCATTCGGC	Tyr 65
R112 F	GACGTGTACACAACTACANNKTATGATGCACCTTCC	Arg112
R112 R	GAAGGTGCATCATAMNNTGTAGTTGTGTACACGTC	Arg112
W129 F	CTACTTTTACGCAGTACNNKAGTGTTCGCCAGTCGAAG	Trp 129
W129 R	CTTCGACTGGCGAACACTMNNGTACTGCGTAAAAGTAG	Trp 129
5-7 F	TAACACAGACTGGTGGCATAATTGGACTGATGG	Tyr 5,Gln7
6-7 F	TAACACAGACTACTTTATCAATCCTACTGATGG	Trp 6,Gln7
5-6-7 F	TAACACAGACTGGTTTCATAATTGGACTGATGG	Tyr 5, Trp 6, Gln7
6-7-9 F	TAACACAGACTACTTTCATAATCCTACTGATGG	Trp 6, Gln7, Trp 9

Table S2 Predicted properties of the novel GH11 xylanase Xyn[st]

Index	Value
Amino acid number	213
Molecular weight	23.48 kDa
Theoretical pI	9.28
Grand average of hydropathy (GRAVY)	-0.450
Signal peptide	SP(Sec/SPI), 28-29
Instability index	22.33

Table S3 Hydrolysis products of xylan analyzed by HPLC

Concentration	Wheat straw xylan		Bagasse xylan	
(mg/ml)	W6F/Q7H	WT	W6F/Q7H	WT
X	0.015 (0.6%)*	0.020 (1%)	/	/
X2	0.441 (19.1%)	0.460 (23.8%)	0.383 (13.0%)	0.365 (16.0%)
X3	1.264 (54.8%)	1.028 (53.2%)	1.643 (55.8%)	1.239 (54.2%)
X4	0.417 (18.1%)	0.282 (14.6%)	0.697 (23.7%)	0.557 (24.4%)
X5	0.171 (7.4%)	0.142 (7.4%)	0.222 (7.5%)	0.125 (5.4%)
XOS	2.293	1.912	2.945	2.286
Conversion rate	23.1%	19.3%	29.5%	22.9%

*: The percentage of xylose oligomer is calculated by dividing each oligomer by sum of xylose (X), xylobiose (X2), xylotriose (X3), xylotetraose (X4), and xylopentose (X5).

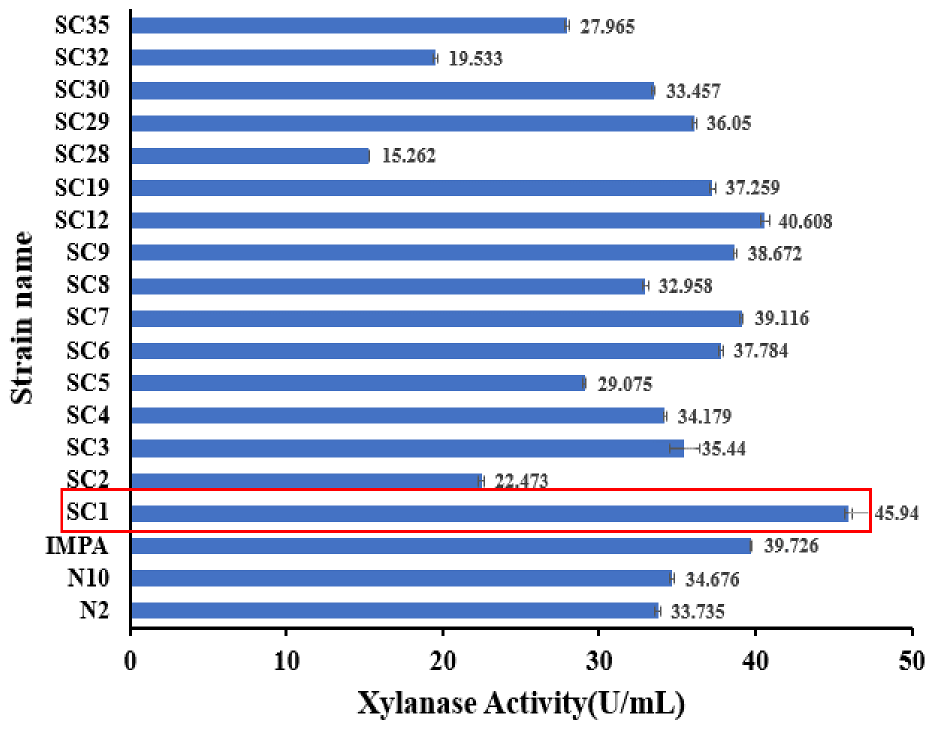

Fig. S1 Preliminary screen of of xylanase secretion by different isolates: xylanase activity was assayed using the culture supernatant with the DNS method. All isolates were propagated to stationary phase and tested in triplicate. The absorbance at 540nm was monitored by a Multimode Reader Spark® (Tecan, Austria).

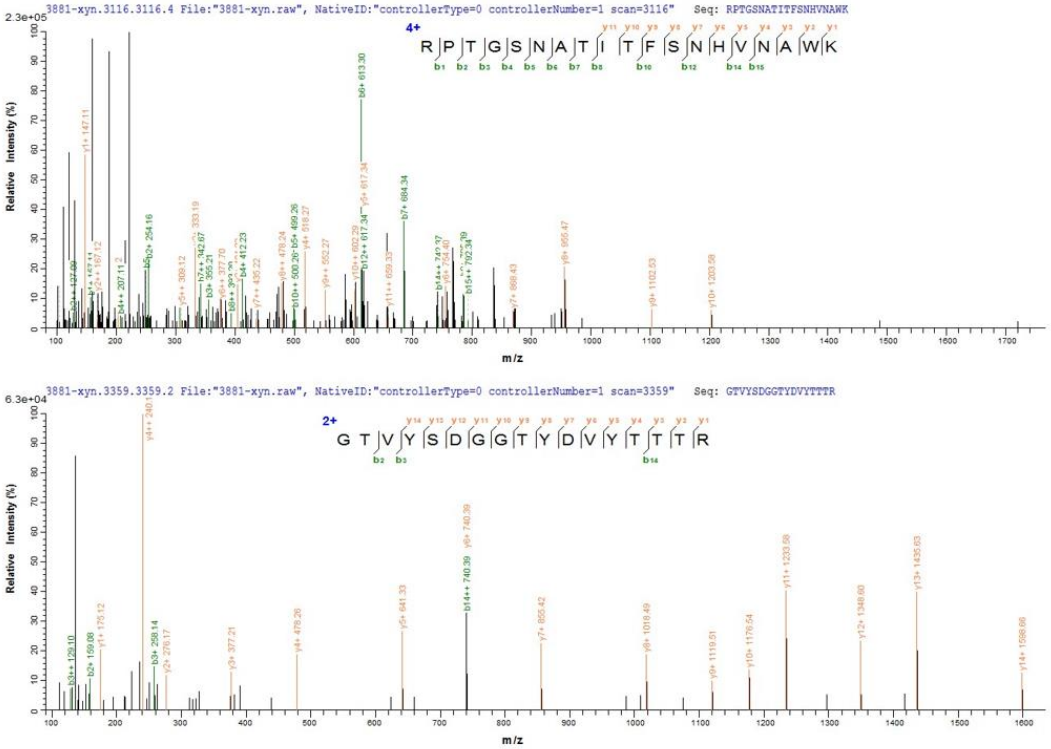

Fig. S2 Xylanase protein identification by LC-MS/MS： The secreted xylanase by *Bacillus sp.* SC1 was precipitated by 65% $(NH_4)_2SO_4$. The target protein band was extracted from sodium dodecyl-sulfate polyacrylamide gel electrophoresis and was identified by liquid chromatography coupled with mass spectrometry

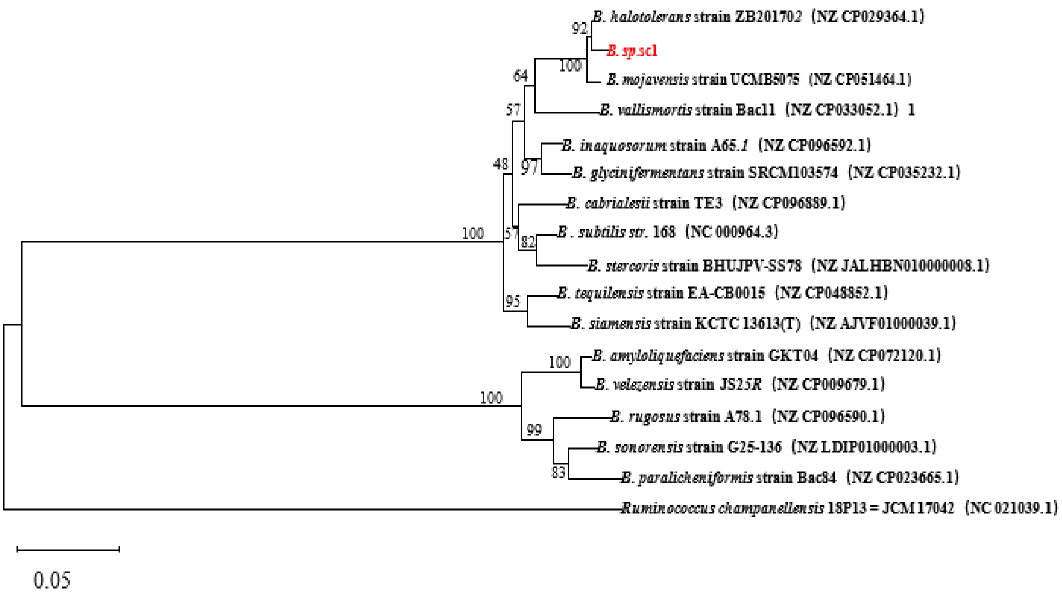

Fig. S3 Phylogenetic tree of Xynst gene from *Bacillus sp.* SC1： A sequence with the size of 642bp was cloned from SC1 and used for nBLAST. A total of 15 representative sequences from different *Bacillus* species and a non-related strain were used for construction of the phylogenetic tree with the neighbor-joining algorithm.

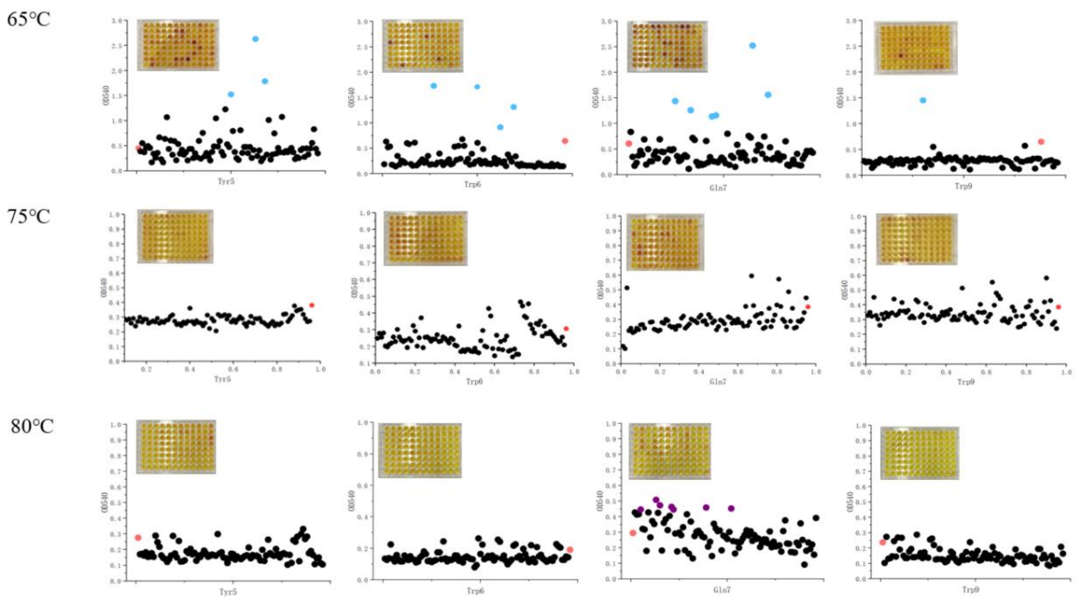

Fig. S4 High throughput screen of SDM library for highly active and thermo-stable mutants: screen of the SDM libraries using 96-well microtitor plates at different temperatures and dot scatter of the absorbance at 540 nm. The red dot represents the wide-type strain that is used as control on each microtitor plate. The blue dots are positive mutants that have significantly higher activities, while black dots are negative or nonsense mutants that have lower or comparable activities than the control.

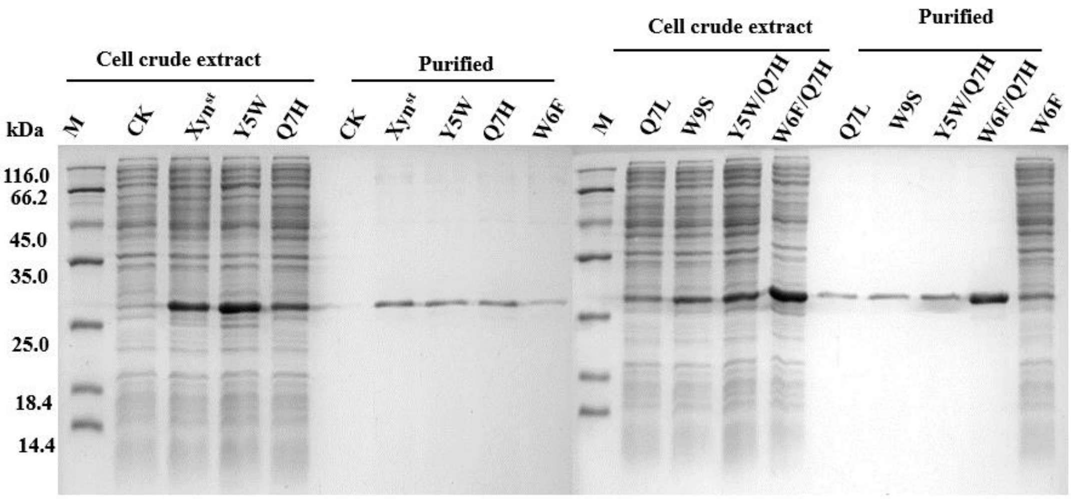

Fig. S5 SDS-PAGE analysis of xylanase expression and purification: The bands with a molecular weight of about 26 kDa are target xylanases. Lane M, protein marker; CK, cell crude extract of *E. coli* BL21/pET-28a-Xyn without induction; Cell crude extract, cell crude extract of *E. coli* BL21/pET-28a-Xyn and mutants induced by IPTG; Purified, purified Xynst or mutant xylanases by affinity chromatography.

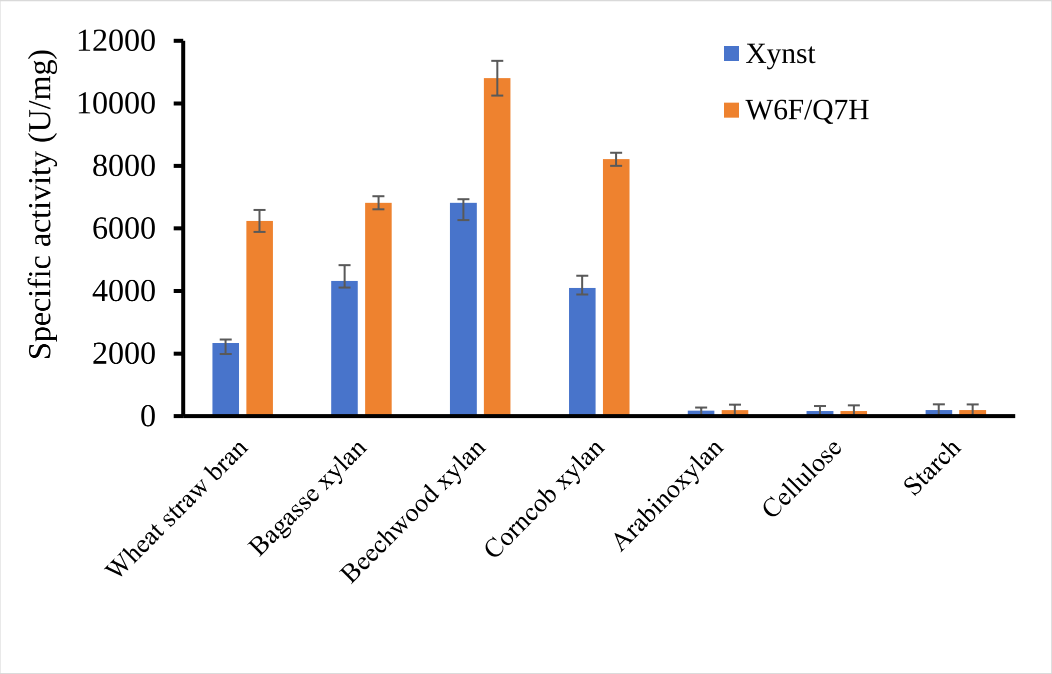

Fig. S6 Substrate preference of xylanase Xynst and W6F/Q7H: different substrates (beechwood xylan, bagasse xylan, borncob xylan, wheat straw xylan, arabinoxylan, cellulose, starch, 1% w/v) in 50mM Gly-NaOH buffer (pH 9.0) were used for reaction with pure recombinant enzymes at 55°C.

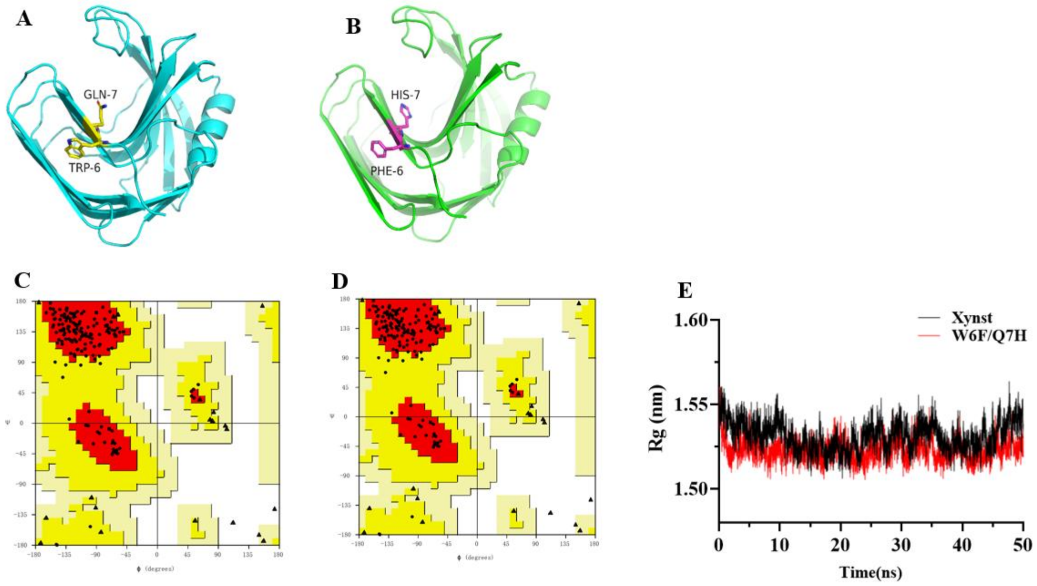

Fig. S7 Molecular dynamics simulations: A and B, the predicted 3D model of Xynst and W6F/Q7H; C and D, the ramachandran plot of Xynst and W6F/Q7H; E, the Rg values of Xynst and W6F/Q7H.

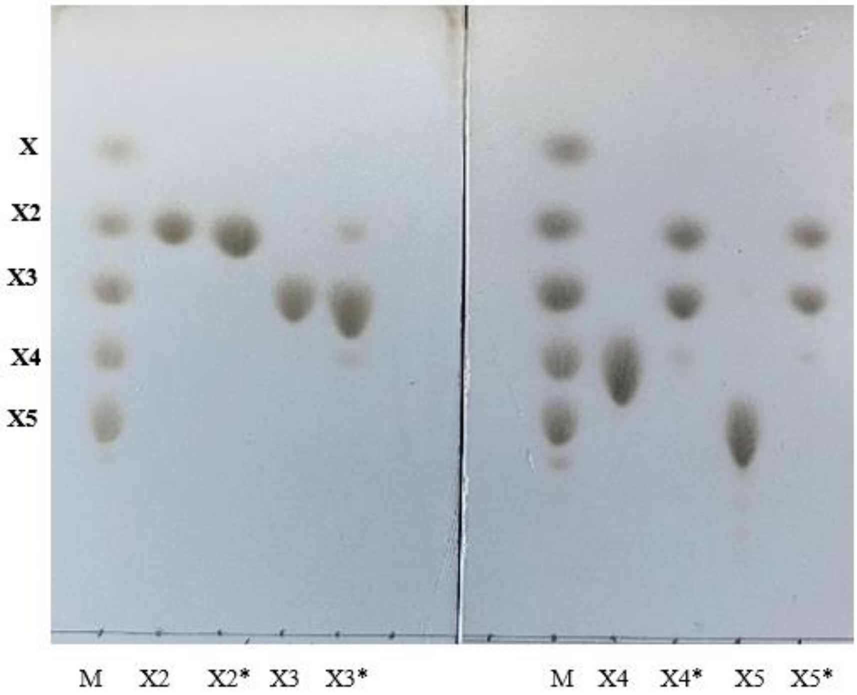

Fig. S8 TLC analysis of the enzymatic degradation by W6F/Q7H: The standards of X2, X3, X4, and X5 were used as substrates, respectively. Enzymatic reaction was carried out at 60℃ for 6h. Lane M: xylooligosaccharides (XOS) standards; X, xylose; X2, xylobiose; X3, xylotriose; X4, xylotetraose; X5, xylopentose; *, post-hydrolysis
